# Insecticidal counting dataset based on one solar insecticidal lamp and two cameras

**DOI:** 10.3389/fpls.2022.995118

**Published:** 2022-10-18

**Authors:** Kai Huang, Lei Shu, Kailiang Li, Yuyu Feng, Zitian Jiang, Yan Zhu

**Affiliations:** ^1^ National Engineering and Technology Center for Information Agriculture (NETCIA), Nanjing Agricultural University, Nanjing, China; ^2^ College of Artificial intelligence, Nanjing Agricultural University, Nanjing, China; ^3^ School of Engineering, College of Science, University of Lincoln, Lincoln, United Kingdom; ^4^ College of Agriculture, Nanjing Agricultural University, Nanjing, China

**Keywords:** insecticidal counting, Solar Insecticidal Lamp (*SIL*), Pulse Number of Insecticidal Discharges (*PNID*), Pulse Number of Insecticidal Sounds (*PNIS*), video data

## 1 Introduction

The principle and period for killing the agricultural migratory pests by Solar Insecticidal Lamp (*SIL*) are as follows: 1) The principle is that the migratory pests are attracted by the *SIL* because of their phototaxis and are killed or seriously injured by high-voltage grid discharge while contacting the metal mesh. 2) The period is mainly from the pest maturity period with flying ability to the pests pre-fertility period with mating and spawning ability (Li et al., 2019). After the pests are killed, insecticidal counting is performed simultaneously to calculate the pests density, further guiding the famers to spray pesticide accurately. It is meaningful that it will reduce the usage of pesticides, thus avoiding serious pollution and damage to the natural ecological environment.

There are various insecticidal counting methods based on *SIL* (Guo, 2015; Wang et al., 2015a; Wang et al., 2015b; Xu and Zhou, 2015; Wang et al., 2016; Li, 2018).When the pests collided with metal mesh, short-circuit of the metal mesh occurred, and the pests were killed by discharge of the mesh. This will cause the fluctuating voltage of both the input and output terminals of the module in the insecticidal lamp, and the module’s function is that DC power supply is converted into high voltage. By detecting the fluctuation of voltage, insecticidal counting can be realized. However, during the working period, a pest sticks to the metal mesh when the pest contacts the metal mesh, leading to the continuous discharge of the mesh. The fact that one pest is killed cannot be reflected just by monitoring the voltage change described above. On the contrary, it will be considered that many pests are killed, leading to inaccurate insecticidal counting.

When a pest is killed by one discharge, the input voltage of the DC boost module changes once, generating a discharge pulse and making the sound “pa”. When a pest is killed by continuous discharge, multiple discharge pulses are generated and continuous sound “pa pa pa…” is made, which is not as loud as the sound “pa”. Although the sound of discharge has such remarkable difference, there is no related research on the accurate insecticidal counting by the sound of discharge at present.

With the emergence of Solar Insecticidal Lamps Internet of Things (*SIL-IoTs*), pests are killed by Solar Insecticidal Lamps (*SIL*) more accurately and efficiently, further realizing real-time report and accurate prediction of pests situation (Li et al., 2019). Combined with the above insecticidal counting analysis of *SIL*, we have modified the testbed, enabling us to acquire the dataset including Pulse Number of Insecticidal Sounds (*PNIS*), Pulse Number of Insecticidal Discharges (PNID), and video data during the discharging process. And the dataset can accurately reflect the quantity of pests being killed.

Scientific value and potential usage of the dataset: Establish the relevant insecticidal counting model to accurately describe the *insecticidal quantity* by machine learning, and further describe the pests population density in the area where *SIL* are deployed. After finishing the insecticidal counting, the *insecticidal quantity* at different times can further facilitate the research on *SIL* energy management, including the time and duration of turning on and off the lamp.

## 2 Methods

**Figure 1:**
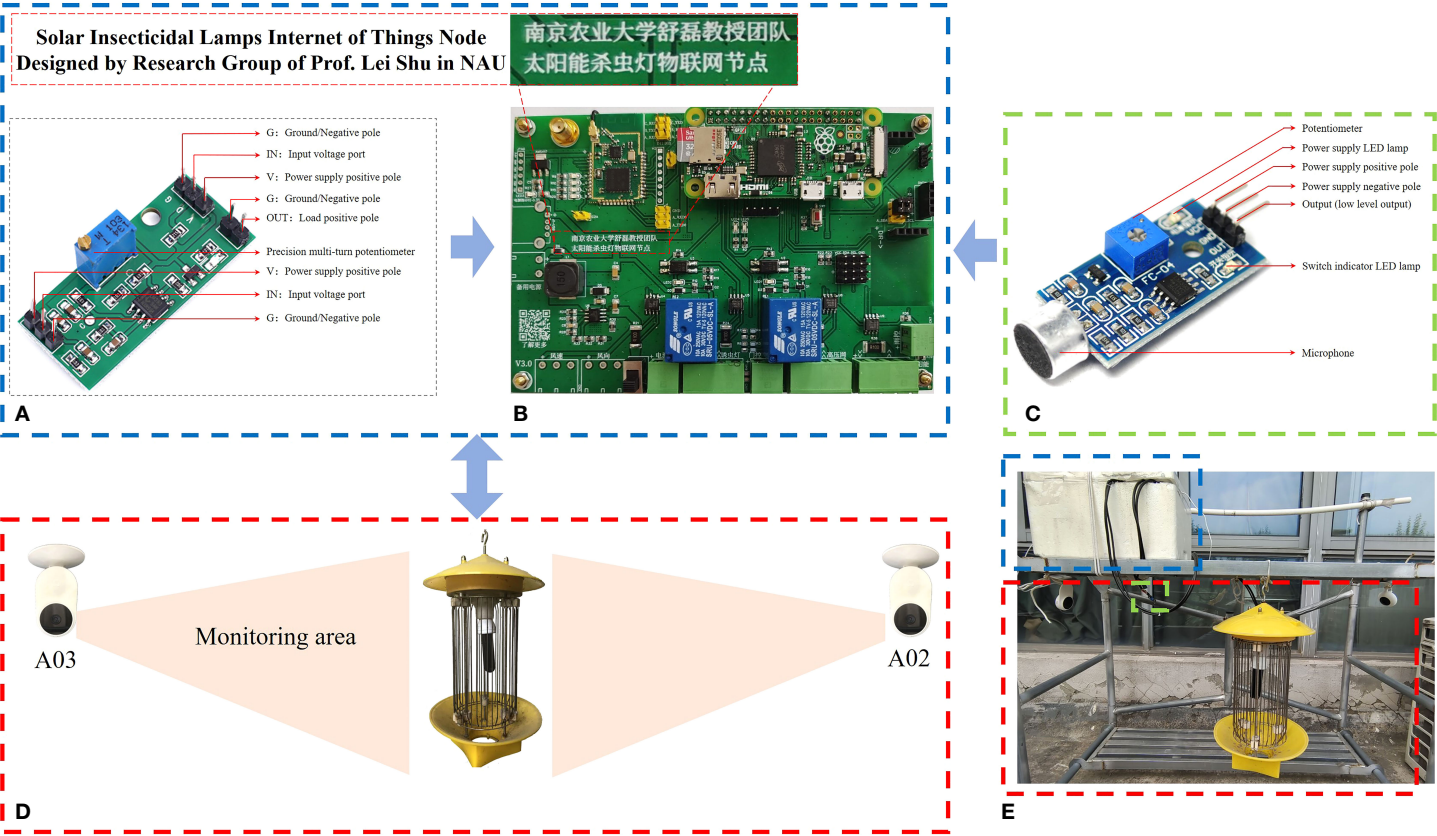
LM393 voltage comparator module **(A)**, Printed Circuit Board (PCB) **(B)**, Risym sound sensor module **(C)**, and insecticidal lamp monitored by two cameras **(D)** are the major components of the testbed **(E)**.

In **Figure 1E**, the testbed was deployed at the open balcony of Yuxian Building in No.40 Dianjiangtai Road, Pukou District, Nanjing, Jiangsu Province of China. The insecticidal lamp was turned on at 19:00 every night and turned off at 4:00 in the next morning from 8/1/2021 to 10/15/2021. The cylindrical metal mesh of the insecticidal lamp was 0.297m high and 0.168m outside diameter, then the size of pests can be calculated through the photos pictured when the pests collided with the metal mesh. When the insecticidal lamp is turned on, the light trapped pests, whose wavelength is 365nm. When the pests flied around the insecticidal lamp, they collided with the metal mesh suddenly, generating a sound “pa”. The *PNIS*, the number of nearby sounds reaching the threshold value of Risym sound sensor module, was acquired through the following process: when the microphone vibrated due to the nearby sounds, the microphone outputted a weak current, whose corresponding voltage was amplified and compared with the reference voltage of power supply; if it was higher than the reference voltage, the *PNIS* plus one. The pests were killed by high voltage (~2150V to ~6000V) pulse current, and the *PNID*, the number of input voltage of metal mesh reaching the threshold value of LM393 voltage comparator module, was acquired through the following process: the input voltage of metal mesh and reference voltage of power supply were compared; if the input voltage was higher than reference voltage, the *PNID* plus one. Both the *PNIS* and *PNID* were acquired every five seconds and were stored in the SD card of Raspberry pi on the PCB in **Figure 1B**. The testbed was equipped with two cameras (Brand: Xiaomi; Type: SXJ02ZM; Resolution: 1080P) in **Figure 1D**, and the working status of the insecticidal lamp was monitored and recorded. Then the collected video data was used to label the data and produce the dataset.

The threshold value of both *PNIS* and *PNID* were adjusted by the potentiometer, making the dataset reflect the insecticidal process.

For LM393 voltage comparator module in **Figure 1A**,

1) The reference voltage is adjusted by the precision multi-turn potentiometer. The voltage generated by both the sensor and the voltage divider resistor is compared with the reference voltage, further realizing the output of the *high levels* and *low levels*.
2) When the input voltage exceeds the reference voltage set by the potentiometer, the OUT terminal outputs a *high level*, and the number of *high levels* increases by one. Each recorded value of *PNID* is the accumulated number of *high levels* within each five seconds.
3) Adjust the presetting threshold by potentiometer, and observe the *PNID* to determine whether the presetting threshold is appropriate.

For Risym sound sensor module in **Figure 1C**,

1) Risym sound sensor module can be used to detect ambient sound intensity.
2) When the ambient sound intensity does not reach the presetting threshold, the OUT terminal outputs a *low level*. When the ambient sound intensity exceeds the presetting threshold, the OUT terminal outputs a *high level*, and the number of *high levels* increases by one. Each recorded value of *PNIS* is the accumulated number of *high levels* within each five seconds.
3) Adjust the presetting threshold by potentiometer, and observe the *PNIS* to determine whether the presetting threshold is appropriate.

## 3 Description of dataset

A total data of fifty-two days was collected by the above testbed from 8/18/2021 to 10/10/2021. Due to rain and testbed failure, the actual number of days with valid data was forty-one days. Since 9/29/2021, Camera A03 was broken due to rain. So there was no video data from Camera A03 after 9/29/2021, which did not affect the integrity of dataset.

**Figure 2:**
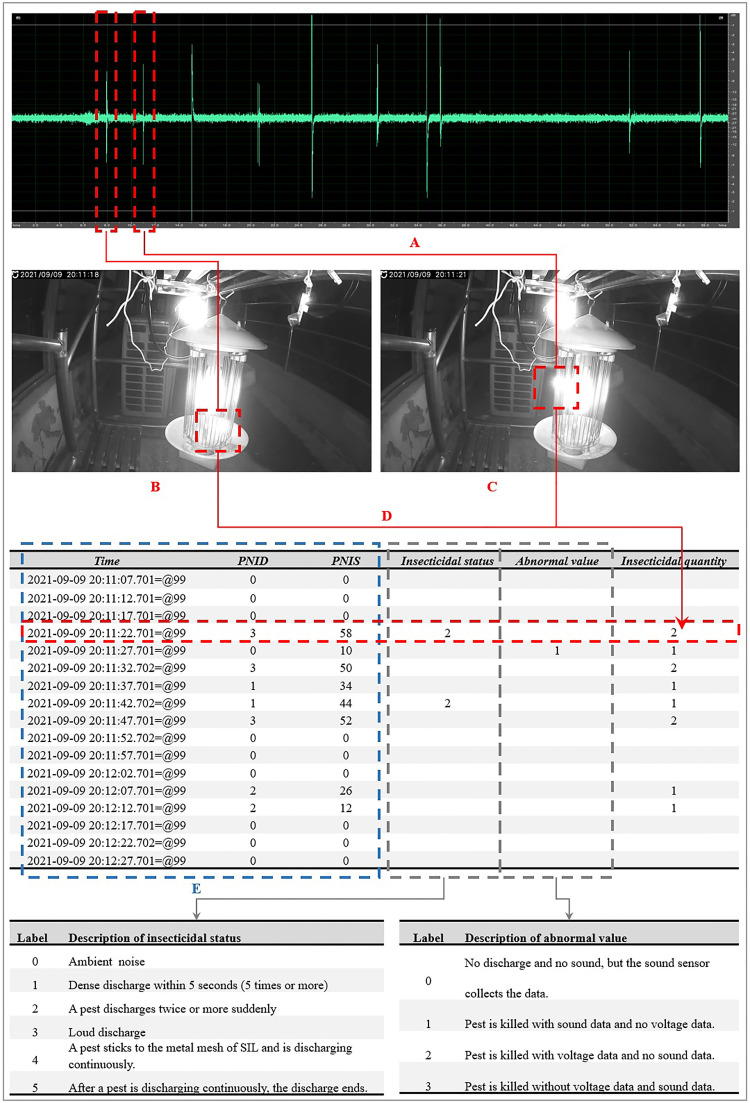
Sound wave was extracted from the video data (9/9/2021 20:11:10 - 9/9/2021 20:12:10) **(A)**, and two screen shots **(B, C)** were captured, indicating that two insects were killed by discharge **(D)**. Then the insecticidal quantity was labeled as “2” in the dataset, in which the time, *PNID*, and *PNIS* were the original data acquired from the testbed **(E)**.

As shown in **Figure 2A**, the sound wave was extracted from the video data (9/9/2021 20:11:10 - 9/9/2021 20:12:10), and the sound wave with two red squares were the sounds when the pests were killed by discharge. Both **Figures 2B, C** depicted the two screen shots of the video data, indicating that two insects were killed by discharge in **Figure 2D**. Then we can add more information based on the video data. Finally, there were six parts in the dataset including *time*, *PNID*, *PNIS*, *insecticidal status*, *abnormal value*, and *insecticidal quantity*.

1) *Time*: The *time* represented the moment when data recording was stopped, and the period for collecting data was the time difference between the previous moment and current moment, which was set as five seconds.
2) *PNID*: The value of *PNID* was the number of *high levels* within five seconds.
3) *PNIS*: The value of *PNIS* was the number of *high levels* within five seconds.
4) *Insecticidal status*: The *insecticidal status* represented how the pests were killed. If a pest was killed by only one discharge, the *insecticidal status* was not labeled.
5) *Abnormal value*: The *abnormal value* was the abnormal response of the sensor in the working time of the testbed.
6) *Insecticidal quantity*: The *insecticidal quantity* the number of pests killed in 5 seconds.
In the dataset, time, *PNID*, and *PNIS* were the original data acquired from the testbed in **Figure 2E**; *insecticidal status*, *abnormal value*, and *insecticidal quantity* were labeled according to the collected video data.

According to the video data, the *insecticidal status* can be divided into six different situations as follows:

1) Label 0: The *PNIS* was not zero in the dataset while no pest was killed, so the microphone collected ambient noise.
2) Label 1: One or more pests were killed by discharge within five seconds, and the number of discharges was five or more.
3) Label 2: When a pest collided with the metal mesh, the pest discharged twice or more suddenly.
4) Label 3: When a pest was killed, the sound of discharge was very loud.
5) Label 4: When a pest stuck to the metal mesh, the mesh was discharging continuously, with the total discharge time exceeding five seconds or even up to several hours. When discharge process lasted for several hours, the situation was that a big pest got stuck on the metal mesh and the mesh was continuously discharging until the water content of the pest dried up, or until the pest fell off the metal mesh.
6) Label 5: After a pest was discharged continuously, the discharge ended within five seconds.

According to the video data, the *abnormal value* can be divided into four different situations as follows:

1) Label 0: Although the sound sensor module acquired *PNIS*, it could be seen from the video data in which no pest was killed.
2) Label 1: A pest was killed by discharge, and the sound sensor module acquired *PNIS*, but the voltage sensor module did not acquire *PNID*.
3) Label 2: A pest was killed by discharge, and the voltage sensor module acquired *PNID*, but the sound sensor module did not acquire *PNIS*.
4) Label 3: A pest was killed by discharge, but both the voltage sensor module and sound sensor module did not acquire data.

After introducing the meaning of each type of data in the dataset in detail, the following two record data in the dataset were taken as examples to illustrate the insecticidal process.

1) For the record data “2021-09-09 20:11:22.701=@99”, the ending time point was 20:11:22.701 on September 09, 2021, and the data recorded in five seconds was from 20:11:17.701 to 20:11:22.701. The *PNID* was 3 and the *PNIS* was 58. In the video data, a pest collided with the metal mesh, the pest discharged twice suddenly at 20:11:18; and another pest discharged at 20:11:21. The *insecticidal status* was labeled as 2, and the *insecticidal quantity* was 2. Both the voltage sensor module and the sound sensor module acquired data, and there was no *abnormal value*, so the *abnormal value* was not labeled.
2) For the record data “2021-09-09 20:11:27.701=@99”, the ending time point was 20:11:27.701 on September 9, 2021, and the data recorded in five seconds was from 20:11:22.701 to 20:11:27.701. The *PNID* was 0 and the *PNIS* was 10. In the video data, it was found that a pest was killed by discharge within five seconds, and there was no corresponding situation of the existing label in the *insecticidal status*, so it was unnecessary to label it. The *PNID* was 0, indicating that the voltage sensor module did not acquire data. If both *PNID* and *PNIS* were 0, there was no need to label. In this dataset, there was no pest killed when the values of both *PNID* and *PNIS* were 0.

## 4 Potential use

The dataset can be used for a variety of methods related to the research of insecticidal counting.

## Data availability statement

Publicly available datasets were analyzed in this study. This data can be found here: https://ieee-dataport.org/documents/insecticidal-counting-dataset-based-one-solar-insecticidal-lamp-and-two-cameras.

## Author contributions

KL and KH participated in the design of the testbed. KH and YF carried out the experiment. YF labeled the data based on the video data. KH, YF, ZJ, LS, and YZ wrote the paper and revised it. All authors contributed to the article and approved the submitted version.

## Funding

This work was supported in part by the National Natural Science Foundation of China under (Grant 62072248), and by the Jiangsu Agriculture Science and Technology Innovation Fund under [Grant CX(21)3060].

## Conflict of interest

The authors declare that the research was conducted in the absence of any commercial or financial relationships that could be construed as a potential conflict of interest.

## Publisher’s note

All claims expressed in this article are solely those of the authors and do not necessarily represent those of their affiliated organizations, or those of the publisher, the editors and the reviewers. Any product that may be evaluated in this article, or claim that may be made by its manufacturer, is not guaranteed or endorsed by the publisher.
